# Perioperative stroke during carotid endarterectomy: benefits of multimodal neuromonitoring - a case report

**DOI:** 10.1186/s12883-022-02835-7

**Published:** 2022-08-31

**Authors:** D. M. Michels, L. C. Van Dijk, D. L. J. Tavy

**Affiliations:** 1 Department of Neurology, Haga Teaching Hospital, Els Borst-Eilersplein 275, 2545 AA The Hague, The Netherlands; 2 Department of Radiology, Haga Teaching Hospital, The Hague, The Netherlands

**Keywords:** Carotid endarterectomy, Monitoring, Electroencephalography, Transcranial doppler, Near-infrared spectroscopy, Case report

## Abstract

**Background:**

Carotid endarterectomy is routinely performed after ischemic stroke due to carotid stenosis. Perioperative, cerebral blood flow and oxygenation can be monitored in different ways, but there is no clear evidence of a gold standard and a uniform guideline is lacking. Electroencephalography and near-infrared spectroscopy are among the most frequently used methods of neuromonitoring. Clinicians should be aware of their pitfalls and the added value of transcranial doppler.

**Case presentation:**

We present the case of an 85-year old male with perioperative haemodynamic stroke during carotid endarterectomy. Ischemic stroke was caused by suddenly increased carotid stenosis resulting in major neurologic deficit. This was registered only by transcranial doppler, while surface electroencephalography and near-infrared spectroscopy failed to detect any significant change in cerebral perfusion, despite a large perfusion defect on computed tomography. Circulation was restored with endovascular treatment and neurologic deficit quickly resolved.

**Conclusion:**

We strongly advocate the practice of multimodal neuromonitoring including transcranial doppler whenever possible to minimize the risk of persistent neurologic deficit due to perioperative stroke during carotid endarterectomy.

## Background

Carotid endarterectomy (CEA) for the treatment of significant carotid stenosis after ischemic stroke is routinely performed worldwide. The risk of perioperative complications such as death or disabling stroke ranges from 3 to 7.5%, particularly during clamping of the carotid artery [1]. Therefore, different methods of monitoring cerebral blood supply and oxygenation have been developed, such as electroencephalography (EEG), transcranial doppler (TCD), stump pressure measurement, near-infrared spectroscopy (NIRS) or somatosensory evoked potentials (SSEP) [2, 3]. However, no conclusive data exist on the optimal combination of monitoring modalities to maximize sensitivity for perioperative cerebral ischemia [2].

## Case presentation

**Fig. 1:**
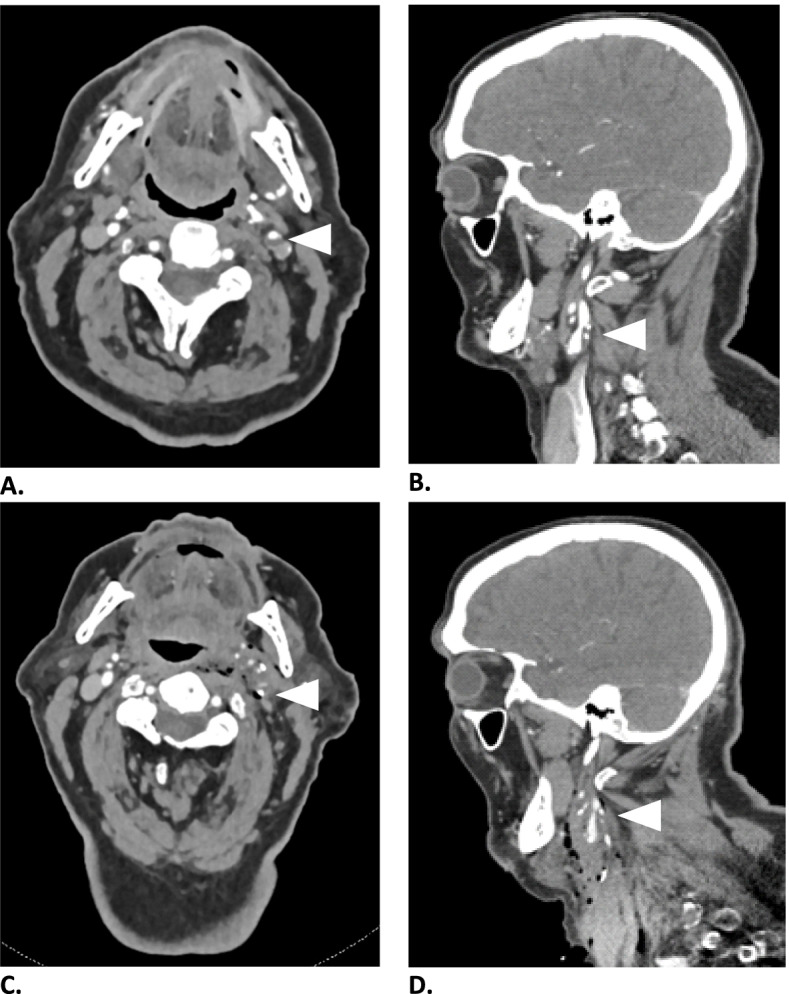
Pre- and postoperative contrast enhanced computed tomography of left ICA stenosis (white arrow). **A** Pre-operative axial view, graded 60–70% using NASCET criteria. **B** Pre-operative sagittal view. **C** Postoperative near occlusion of the left ICA with postoperative subcutaneous emphysema, axial view. **D** Postoperative sagittal view

We describe the case of an 85-year old, right-handed male with a history of myocardial infarction and peripheral vascular disease. He underwent elective CEA after suffering a minor stroke of the left middle cerebral artery (MCA) territory due to an ipsilateral 60–70% internal carotid artery (ICA) stenosis according to North American Symptomatic Carotid Endarterectomy Trial (NASCET) criteria (Fig. 1). There was no significant contralateral stenosis. Preoperative carotid ultrasound confirmed the 60–70% stenosis and showed no unfavourable plaque characteristics such as instability or adherent thrombus.

**Fig. 2:**
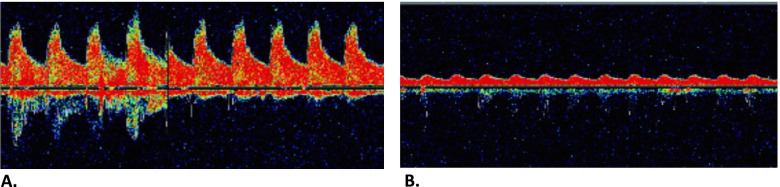
Perioperative transcranial doppler (TCD) signal of the left middle cerebral artery. **A** Before dissection, mean velocity 70 cm/s. **B** During dissection, mean velocity 13 cm/s (> 80% decrease compared to the start of procedure)

At the time of surgery almost 2 weeks later, only minimal neurologic deficit remained. Surgery was performed under general anaesthesia and perioperative monitoring was performed with visual and quantitative EEG, TCD of the left MCA, and NIRS of both hemispheres. During dissection but before clamping of the common and internal carotid artery, a sudden and severe decrease in mean flow velocity of the left MCA was observed (70 to 13 cm/s, > 80% decrease), while the patient was otherwise hemodynamically stable (Fig. 2). Concurrently, NIRS demonstrated only a slight decrease (7%) in oxygenation of the left hemisphere while the right hemisphere remained stable. Visual and quantitative EEG remained completely unchanged for both left and right hemisphere.

**Fig. 3:**
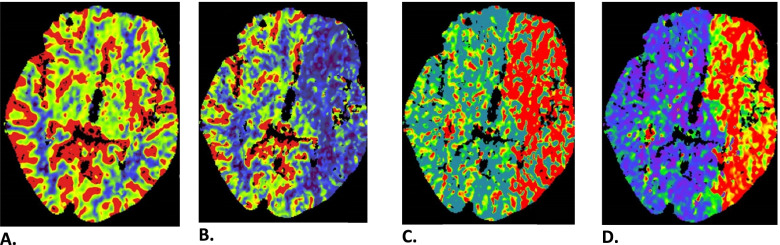
Postoperative computed tomography perfusion imaging showing a large perfusion defect of the entire anterior circulation of the left hemisphere consistent with hypoperfusion due to severe carotid artery stenosis. **A** Cerebral blood volume. **B** Cerebral blood flow. **C** Time to peak. **D** Mean transit time. Decreased cerebral blood flow with normal cerebral blood volume indicate a small infarct core and large penumbra, e.g. salvageable tissue

**Fig. 4:**
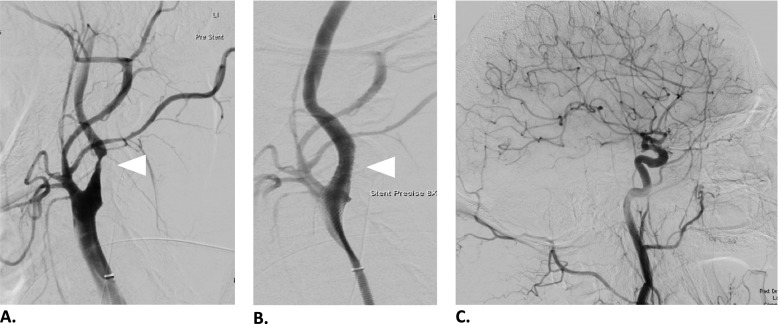
Carotid artery angioplasty and stenting. **A** Left internal carotid artery with near occlusion (white arrow). **B** Restored flow after carotid artery stenting. **C** Angiography post-stenting showing no occlusions of the distal middle cerebral artery territory

Because of a persistent change in TCD and NIRS parameters there was a strong suspicion of cerebral complications. Since neurologic examination was not possible due to general anaesthesia, the decision was made to terminate the procedure. After sedation effects had worn off the patient was found to have a global aphasia and right-sided paralysis. Computed tomography with angiography of head and neck demonstrated a near occlusion of the left ICA (Fig. 1). This may have been due to intraluminal thrombus, or intraplaque hematoma due to dissection. No embolic occlusion of the middle (MCA) or anterior cerebral artery (ACA) was found. Perfusion imaging showed a large perfusion defect of the entire anterior circulation of the left hemisphere compatible with hypoperfusion (Fig. 3). Subsequently, an emergency carotid artery angioplasty with stenting was performed to restore perfusion, since returning to the operating room to finish the CEA was deemed too time-consuming. Angiography after stent placement demonstrated no embolic occlusions of the MCA or ACA territory (Fig. 4). Perfusion was restored 4 h after the perioperative changes in TCD and NIRS occurred. Full recovery of aphasia and right-sided paralysis was observed immediately after stent placement. At 6 months follow-up, no stroke recurrence has been observed.

## Discussion and conclusions

Clinically significant carotid artery stenosis (defined as ≥50–99% stenosis using North American Symptomatic Carotid Endarterectomy Trial (NASCET) criteria) is a major risk factor for ischemic stroke, with recurrence risks after initial stroke of up to 20–30% within 30 days, depending on severity of stenosis, plaque characteristics and general cardiovascular risk factors [4]. CEA has been proven superior to best medical treatment in reducing the risk of death or major disability due to recurrent stroke within 5 years, with an absolute risk reduction of 6.7% [5]. The most important risk of CEA is the development of perioperative stroke, the majority of which are caused by embolism, and around 20% due to hemodynamic changes after clamping of the carotid arteries [6].

Different monitoring modalities have been used to detect cerebral ischemia during carotid artery clamping, necessitating shunting. These include surface EEG, TCD, stump pressure measurement, NIRS, somatosensory and motor evoked potentials, or even awake surgery. However, no national or international guideline on the optimal use of neuromonitoring during CEA exists. Few direct comparisons between available tests have been made, and no studies have yet demonstrated any method to be superior [2].

Awake surgery under regional anaesthesia is the most reliable way to determine neurologic deficit, but can be technically challenging and possibly has a higher risk of complications compared to general anaesthesia [7].

The use of EEG for the detection of hemodynamic changes and perioperative stroke due to clamping has been described in several previous studies [8, 9]. Cerebral hypoperfusion during carotid clamping can be identified by asymmetrically increased delta and theta activity or decrease in fast activity. EEG asymmetry is assessed visually but can also be measured quantitatively via automatically calculated parameters, such as the brain symmetry index, aiding in the detection of minor differences [10]. Diagnostic accuracy for hypoperfusion is generally reported to be good [8, 9], although the severity of neurologic deficit in patients with EEG changes is rarely described. It is therefore remarkable to note that in our case a major perfusion defect accompanied by severe deficit was completely undetected by EEG.

Interestingly, both visual and quantitative EEG assessment showed no sign of asymmetry in our patient, while a significant decrease in flow velocity on TCD was seen, accompanied by a slight decrease in ipsilateral NIRS. It remains unclear why no surface EEG abnormalities were found. This could possibly be explained by collateral circulation via the extracranial and leptomeningeal arteries, preventing cortical ischemia thus preserving synaptic function, which is measured with surface EEG. The observed neurologic deficit could be attributed to ischemia of the basal ganglia and internal capsule, as these areas lack proper collateral circulation. However, CT perfusion did demonstrate widespread cortical perfusion defects. It is possible that at the time of scanning, over 90 minutes after the first change in TCD signal was observed, collateral circulation had already been exhausted.

This case highlights the added benefit of TCD alongside other monitoring methods. EEG, NIRS and SSEP measure indirect parameters of cerebral perfusion, such as electric synaptic function and tissue oxygenation. TCD on the other hand measures actual blood flow, making it more sensitive to minor disturbances in cerebral perfusion due to carotid clamping or changes in plaque morphology. Furthermore, TCD can also be used to detect the passage of micro-emboli during surgery, with may predict postoperative stroke [11]. Unfortunately, a suitable temporal bone window for TCD is not always available, which occurs in up to 20% of patients, particularly in elderly [12]. Furthermore, a 2017 meta-analysis determined the sensitivity of TCD alone for detecting perioperative stroke at only 56% [6]. Therefore, additional monitoring with NIRS can be of value. Several studies have investigated its diagnostic accuracy, using varying cut-off values. One large cohort study found a relative decrease of 13% in NIRS during clamping to have a 100% sensitivity of detecting cerebral ischemia, with EEG serving as reference [13]. Other smaller studies have described more lenient cut-off values, ranging from 20 to 41% [14, 15]. Our case illustrates that even a 7% ipsilateral decrease can be associated with severe neurologic deficit. This is in line with a recent meta-analysis on the diagnostic accuracy of NIRS for cerebral ischemia in awake patients, which found a sensitivity of 72% and specificity of 84% when cut-off values of 9–25.8% were used, leading to the authors’ conclusion that NIRS is not accurate enough to be used as a sole neuromonitoring modality [16].

With various options and no clear gold standard, the type of neuromonitoring used during CEA will often depend on availability and local expertise, and varies both regionally and internationally. Previously, calls for simultaneous implementation of different monitoring methods have been raised [3]. However, a recent study has shown a variety of monitoring strategies used across different centres in The Netherlands, including awake surgery, EEG, TCD, stump pressure measurement and no monitoring due to routine shunting in all patients. Only 56% of the interviewed centres reported the combined use of EEG and TCD [17]. This study indicates that, although the pitfalls of each monitoring method are generally well-known, the use of combined monitoring is not yet common practice. It is also important to note that routine shunting only addresses the risk of haemodynamic complications due to clamping, and provides no safeguard against embolic stroke.

In conclusion, haemodynamic stroke due to sudden changes in plaque morphology during routine CEA is a rare complication, having not previously been reported in literature. Clinicians should be aware of the pitfalls in diagnostic accuracy of monitoring modalities such as EEG, TCD and NIRS. Our case highlights the benefits of multimodal neuromonitoring, which is not yet the standard of care in the Netherlands.

When perioperative stroke is suspected, urgent angiography can be performed to assess treatable intracranial occlusions. If necessary, cessation of the procedure for clinical assessment should be considered, followed by endovascular treatment (thrombectomy and/or carotid artery stenting) when possible.

## Data Availability

Not applicable.

## Abbreviations

ACA: Anterior cerebral artery
CEA: Carotid endarterectomy
EEG: Electroencephalogram
ICA: Internal carotid artery
MCA: Middle cerebral artery
NASCET: North-American symptomatic carotid endarterectomy trial
NIRS: Near-infrared spectroscopy
SSEP: Somatosensory evoked potential
TCD: Transcranial doppler
